# Advancing next-generation brain organoid platforms for investigating traumatic brain injury from repeated blast exposures

**DOI:** 10.3389/fbioe.2025.1553609

**Published:** 2025-06-18

**Authors:** Eyal Bar-Kochba, Catherine M. Carneal, Vanessa D. Alphonse, Andrea C. Timm, Amanda W. Ernlund, Carissa L. Rodriguez, Itzy E. Morales Pantoja, Lena Smirnova, Thomas Hartung, Andrew C. Merkle

**Affiliations:** ^1^ Research and Exploratory Development Department, Johns Hopkins University Applied Physics Laboratory, Laurel, MD, United States; ^2^ Center for Alternatives to Animal Testing (CAAT), Department of Environmental Health and Engineering, Bloomberg School of Public Health, Johns Hopkins University, Baltimore, MD, United States; ^3^ CAAT-Europe, University of Konstanz, Konstanz, Germany

**Keywords:** traumatic brain injury, brain organoids, repeated blast, low-level blast, primary blast, *in vitro* model

## Abstract

Service members and law enforcement personnel are frequently exposed to blast overpressure during training and combat due to the use of heavy weaponry such as large-caliber rifles, explosives, and ordnance. The cumulative effects of these repeated low-level (<4 psi) blast exposures can lead to physical and cognitive deficits that are poorly understood. Brain organoids—human stem cell-derived three-dimensional *in vitro* culture systems that self-organize to recapitulate the *in vivo* environment of the human brain—are a promising alternative biological model to traditional cellular cultures and animal models, offering a unique opportunity for studying the mechanisms of mild blast-induced traumatic brain injury (mbTBI) resulting from repeated exposure. In this article, we review the current state of brain organoid models and discuss future directions for advancing their physiological relevance for studying mbTBI. These will be presented within a framework for developing next-generation platforms that integrate relevant loading devices, as well as non-invasive technologies for assessing the brain organoid’s response while increasing throughput. These next-generation platforms aim to accelerate the development of new interventions for mbTBI.

## 1 Introduction

During training and combat, military and law enforcement personnel are frequently exposed to repetitive low-level blast (rLLB) from heavy weaponry, including artillery, mortars, shoulder-fired weapons, stun grenades, and breaching explosives (Kamimori et al., 2017; Skotak et al., 2019; Thangavelu et al., 2020; Belding et al., 2021; Boutté et al., 2021; Wiri et al., 2023; Gilmore et al., 2024). These blasts can generate overpressures which exceed 90 kPa during military training exercises (Wiri et al., 2023), surpassing the current safety standard of 28 kPa (or 4 psi) based on human tympanic membrane rupture (Hicks, 2024). Compounding this issue, personnel may experience over a hundred low-level blast overpressure events during a single training exercise (Wiri et al., 2023) and many more over the course of their career. While severe blast-induced traumatic brain injury (bTBI) from high-level blast explosions has been extensively investigated (Rosenfeld et al., 2013), growing evidence suggests that rLLB exposure can result in subconcussive or mild blast-induced traumatic brain injury (mbTBI) (Belding et al., 2021; Siedhoff et al., 2022). This form of bTBI is associated with chronic issues, including psychiatric disorders, motor and cognitive impairment, sleep disorders and pain (Siedhoff et al., 2022).

Preclinical models are essential for elucidating the underlying mechanisms of mbTBI, which remain poorly understood. This knowledge gap hinders the advancements in preventative measures (e.g., safe standoff distances, weapons modifications, personal protective equipment (PPE), and prophylactics), diagnostics (e.g., molecular biomarker assays and medical imaging), and treatments (e.g., pharmaceuticals). In the past decade, there has been an increasing number of animal studies that focus on rLLB (Ravula et al., 2022). These studies have provided important insights, revealing pathophysiological changes such as neuroinflammation, axonal damage, and glial activation, as well as behavioral deficits (Ravula et al., 2022). However, animal models face several challenges—such as low throughput, difficulties in generating rLLB exposure with appropriate mechanical boundary conditions, issues with reproducibility, and limited relevance to human neuroanatomy and neurophysiology—all of which are critical considerations when studying mbTBI.

*In vitro* cell culture models have also been extensively used to study bTBI. These models offer the advantage of isolating specific variables that would otherwise confound results. For example, *in vitro* models are easier to manipulate than *in vivo* models and offer the opportunity to independently study the effects of primary (i.e., from pressure wave), secondary (i.e., from tearing), or tertiary (i.e., from inertia or blunt forces) loading mechanisms, which are biomechanically distinct. Additionally, *in vitro* models are more accessible for a broader range of analytical tools, such as advanced imaging modalities, diverse assays for studying molecular pathways such as RNA sequencing and gene editing techniques to identify and manipulate specific genetic factors that influence injury responses and degenerative pathways (Chen et al., 2009; Beltrán et al., 2023; Lai et al., 2024). However, even with these advantages, these models do not capture the complexity of the *in vivo* human brain environment, even with more complex three-dimensional cellular cultures (Cullen et al., 2007; Bar-Kochba et al., 2016; Sawyer et al., 2017; 2018; Snapper et al., 2023; González-Cruz et al., 2024).

Recent advances in the generation of three-dimensional (3D) brain-like structures, called brain organoids, offer immense potential as a new *in vitro* model of the human brain (Smirnova and Hartung, 2024). These brain organoids are differentiated from human induced pluripotent stem cells (iPSCs) and form to resemble the cellular composition, diversity, and architecture of different anatomical regions of the human brain, e.g., midbrain, thalamus, and cerebral cortex (Susaimanickam et al., 2022). Brain organoids mimic key features of the human brain including myelination, synaptic connections, and patterns of gene expression (Vanvliet et al., 2007; Chesnut et al., 2021b; Modafferi, 2021). Functionally, brain organoids have shown spontaneous neural activity and the formation of neural circuits (Trujillo et al., 2019). Due to these unique properties, brain organoids have been used to study various neurodegenerative diseases and neurodevelopmental disorders (Pamies et al., 2017; 2022; Chesnut et al., 2021b; Modafferi, 2021; Eichmüller and Knoblich, 2022; Ravula et al., 2022; Susaimanickam et al., 2022; Smirnova and Hartung, 2024).

Studies have shown that brain organoids are able to recapitulate the key pathological changes associated with various TBI exposures (Zander et al., 2017; Ramirez et al., 2021; Silvosa et al., 2022; Beltrán et al., 2023; Lai et al., 2024). Zander et al. applied explosive blast overpressure waves to brain organoids and found increased formation of reactive oxygen species and membrane permeability (Zander et al., 2017). Silvosa et al. exposed cerebral organoids to pressure waves with varying frequencies and found that higher-frequency pressure resulted in increased apoptosis and network desynchronization (Silvosa et al., 2022). Ramirez et al. embedded cerebral organoids within a surrogate brain placed in a mouse skull and induced injury via controlled cortical impact. One week post impact, they found increased astrogliosis, neuronal damage, and apoptosis, which was similar to paired experiments with mice (Ramirez et al., 2021). In another controlled cortical impact study, Beltrán et al. used RNA sequencing to find genes that regulate inflammation, cell death, and immune dysregulation (Beltrán et al., 2023). Lastly, Lai et al. applied high-intensity focused ultrasound to cortical organoids, revealing tau phosphorylation and TDP-43, which was prominent in deep-layer neurons. Although brain organoids are still an emerging model for TBI research and require significant advancements to enhance their applicability to humans, these studies underscore their potential for investigating TBI (Jgamadze et al., 2020; LaPlaca and Brody, 2022).

**FIGURE 1:**
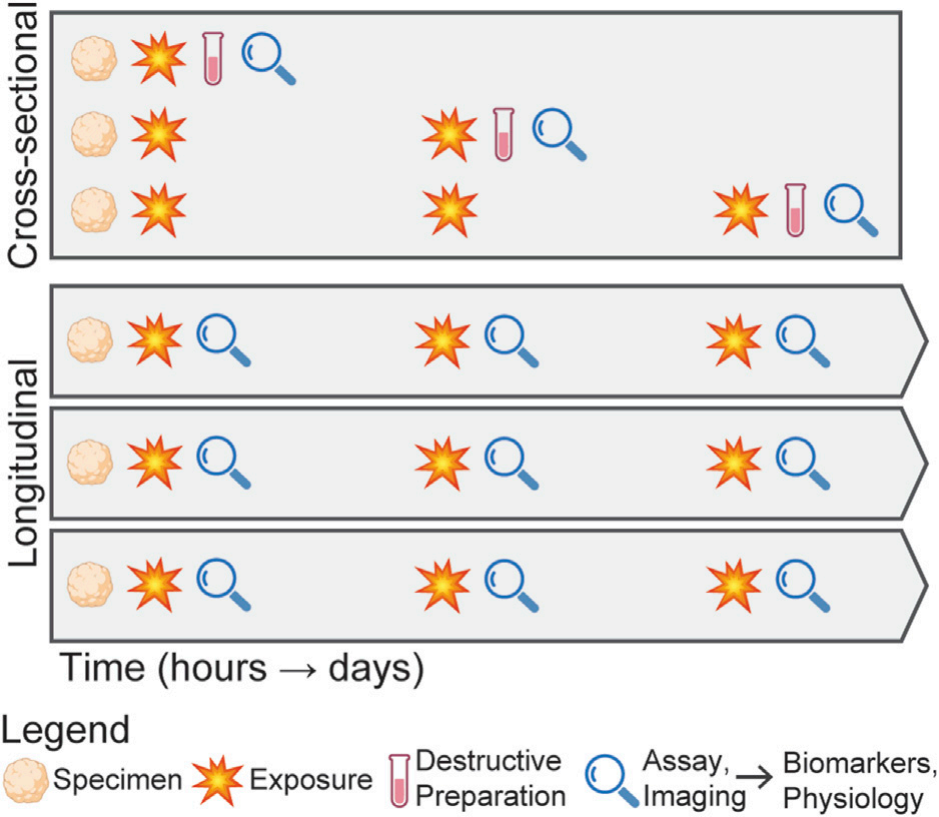
Framework for next-generation mbTBI research platforms, highlighting three core technological areas: (1) loading devices capable of accurately replicating the human brain’s biomechanical conditions, (2) advanced brain organoids that more effectively mimic the *in vivo* environment, and (3) technologies for non-invasively evaluating the biological responses to loading while increasing throughput. This framework aims to accelerate research into determining mbTBI mechanisms, enabling rapid assessment of new interventions.

In this article, we review the current state of brain organoids and present a framework (Figure 1) for developing next-generation platforms tailored to study mbTBI from rLLB. The framework focuses on three key technological areas, each discussed in context of current research:

• Loading devices capable of accurately replicating the biomechanical loading conditions in the human brain during rLLB exposure *in vitro.*
• Advancements in brain organoids to more effectively replicate the *in vivo* environment of the human brain.
• Non-invasive technologies for evaluating biological responses to loading while increasing throughput

The development of these new platforms will accelerate research to elucidate the mechanisms of mbTBI, including determining the dose-response relationship, molecular pathways involved, timelines of injury progression, recovery processes, and functional effects such as learning or memory. This understanding will enable researchers to rapidly evaluate the efficacy of interventions such as prophylactics and therapeutics.

## 2 Study design considerations

**FIGURE 2:**
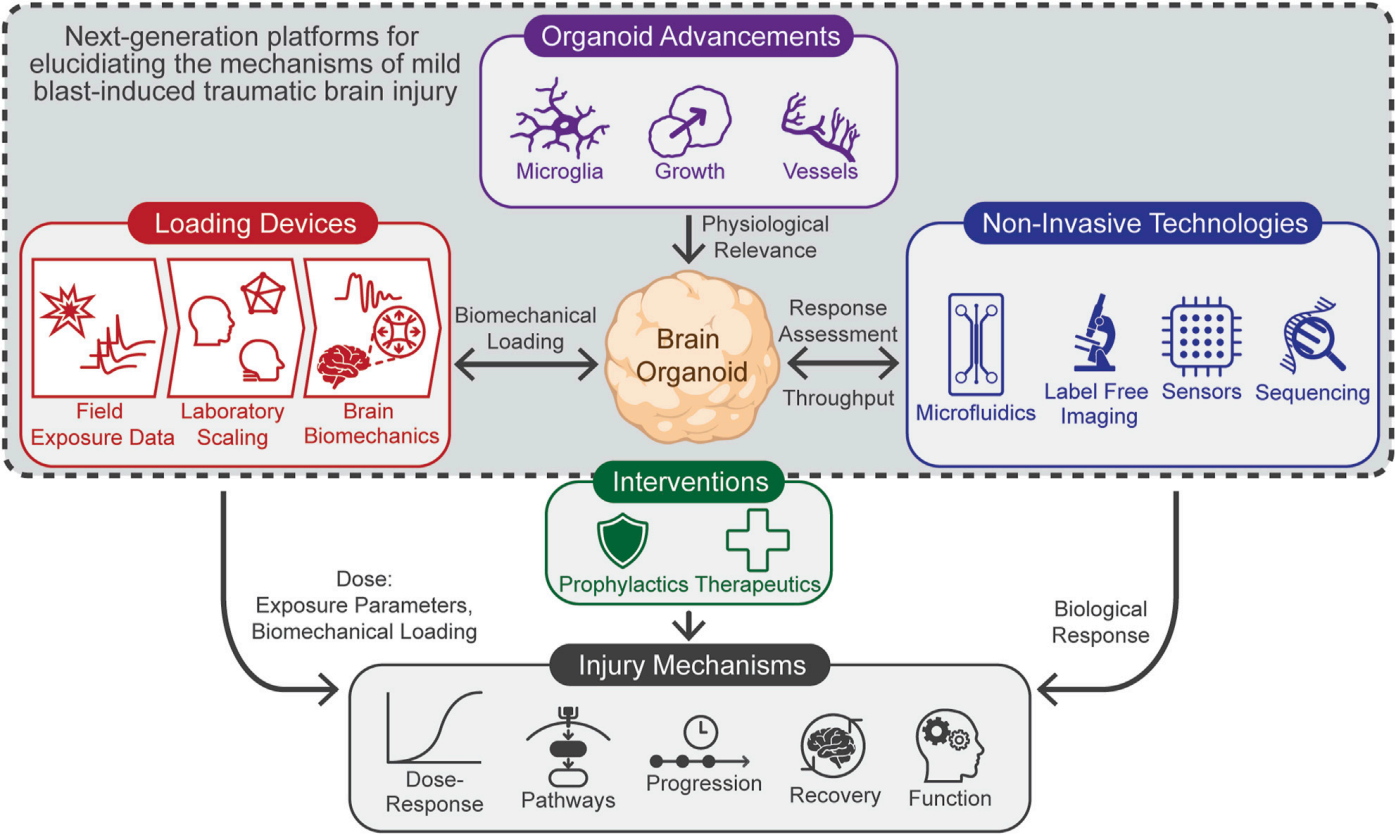
Schematic comparing cross-sectional and longitudinal experimental designs for studying mbTBI from repeated exposures. In the cross-sectional design, specimens are exposed to a blast condition, then destructively processed and analyzed at discrete time points, requiring three organoids to evaluate three post-exposure time points. In contrast, the longitudinal design involves exposing the same specimen to repeated blasts and continuously evaluating injury progression without destructive processing, resulting in more replicates for a single exposure condition.

In studying mbTBI, it is critical to consider the overall experimental design as the injury response is not immediate (Hernandez et al., 2018), making such studies both time-intensive and costly. Further complicating these studies, both the pressure waveform characteristics (e.g., peak pressure and duration) and interval between exposures affect the biomechanical environment in the brain during rLLB exposure. Cross-sectional designs, where the specimen is loaded and then destructively processed and analyzed (Figure 2), for example, with bulk RNA sequencing or immunohistochemistry, provide a detailed snapshot of the injury state, but fail to capture the temporal dynamics of the injury progression. In contrast, longitudinal designs enable non-invasive evaluation of the biological responses, which reduces inter-subject variability and enables the assessment of injury progression within a single specimen, making it better suited for exploring a larger range of loading parameters. Despite these advantages, longitudinal designs face challenges due to the limited availability of techniques capable of non-invasive measurements. However, emerging technologies, including microfluidic systems (Zhao et al., 2024), label-free imaging approaches (Keshara et al., 2022), molecular assays (Abdollahi, 2021), and sensors (Kang et al., 2024), are beginning to address these limitations, making longitudinal designs increasingly feasible.

## 3 Loading devices

A platform for studying mbTBI must effectively tease apart the complex relationship between exposure and injury response (LaPlaca and Brody, 2022). *In vitro* devices must methodically apply controlled loading to understand this complex relationship (LaPlaca and Brody, 2022); however the loading parameters need to accurately reflect the range of exposures experienced in real training and combat scenarios. Characterizing this range requires scaling externally measured field exposures (Kamimori et al., 2017; Skotak et al., 2019; Thangavelu et al., 2020; Wiri et al., 2023) to the biomechanical environment in the brain experimentally (Elster et al., 2023) or *in silico* (Gupta and Przekwas, 2013; Gupta et al., 2017), which are then applied as loading conditions to brain organoids. In this section, we review the biomechanics of blast loading to the head and approaches for scaling these loads to *in vitro* devices.

### 3.1 Characterizing the brain biomechanics

Primary bTBI occurs when a blast pressure wave propagates through the skull, loading the brain tissue directly. In contrast, secondary injuries from shrapnel, and tertiary injuries from rapid accelerations are typically associated with moderate to severe blast exposures (Rosenfeld et al., 2013). These secondary and tertiary effects are less relevant for LLB (Säljö et al., 2008; Rosenfeld et al., 2013), making the primary blast pressure response crucial to replicate experimentally when studying mbTBI.

The brain biomechanics underlying mbTBI are complex due to the interaction of the incident shockwave with the heterogeneous structures and mechanical properties of the human head (Liang et al., 2021). Across a variety of models, including post-mortem human subjects (Bir, 2011; Ganpule et al., 2013; Ott et al., 2013; Ouellet et al., 2014; Iwaskiw et al., 2018), *in silico* (Moore et al., 2009; Taylor and Ford, 2009; Nyein et al., 2010; Chafi et al., 2011; Panzer et al., 2012; Tan et al., 2017; Tan and Matic, 2020; Li et al., 2024), and anthropometric surrogates (Merkle and Carneal, 2012; Hua et al., 2014; Ouellet and Philippens, 2018), the skull acts as a filter, attenuating the high-frequency delta response of the incident shockwave. The transmitted pressure wave propagates through the brain tissue, undergoing multiple reflections within the intracranial cavity due to acoustic impedance mismatches between the cranium and brain (Gupta et al., 2021; Liang et al., 2021). These reflections result in intracranial pressure (ICP) wave interference and oscillatory characteristics with multiple peaks in the 0.5–10 kHz frequency range that persist for 1–10 m.

A full characterization of the tissue-level stress or strain state is challenging since deviatoric stresses in experimental studies are not measured due to sensor limitations. However, computational models have reported deviatoric stresses that are over 100 times lower than pressure (Taylor and Ford, 2009), which is attributable to the high bulk-to-shear modulus ratio of brain and it’s confinement within the intracranial cavity. Deviatoric stresses also persist milliseconds after the initial ICP wave (Taylor and Ford, 2009), suggesting they result from brain strains and displacements (Iwaskiw et al., 2018) caused by overall kinematic head motion, which is less relevant for LLB (Säljö et al., 2008; Rosenfeld et al., 2013). Elster et al. presents a comprehensive review of the experimental studies that measure the brain biomechanics during blast exposures (Elster et al., 2023). However, a comprehensive understanding of the brain biomechanics is challenging due to it being dependent upon many factors, including incident shockwave direction (Li et al., 2024), surface reflections (Tan et al., 2017), use of PPE (Moss et al., 2009; Nyein et al., 2010; Alphonse et al., 2020; Elster et al., 2023; Li et al., 2024), and anthropometric variations.

With recent field studies monitoring rLLB exposure (Kamimori et al., 2017; Skotak et al., 2019; Thangavelu et al., 2020; Wiri et al., 2023), there are opportunities to replicate these conditions experimentally or *in silico* to characterize the brain biomechanics. Critical to these studies is addressing the variations in exposures associated with large diversity of weaponry (Wiri et al., 2023), including the potential cumulative effects of automatic weapons with firing rates that approach the duration of ICP waves, potentially resulting in rapid ICP accumulation.

Additionally, several challenges related to spatiotemporal scales must be addressed when translating results between experimental and computational models, particularly when considering the differences between organoid models and the human brain. At the continuum level, the brain’s mechanical properties behaves as a nonlinear viscoelastic material that is highly rate-dependent (Procès et al., 2022). Therefore, characterizing and assigning these properties in computational models becomes difficult at the time scales of blast exposure. At the cellular scale, deformations are spatially heterogeneous due to the cell-extracellular matrix and cell-cell interactions, resulting in strain concentrations at micro-interfaces with impedance mismatches (Nakagawa et al., 2011), such as synapses (Gharahi et al., 2023). To accurately model these micro-interfaces, advancements in mechanobiology models that bridge continuum and molecular scales are essential (Montanino et al., 2020; Gharahi et al., 2023).

### 3.2 Load scaling to *in vitro* devices

Scaling the effective primary blast loading from an explosive event to an *in vitro* device is challenging. Various systems have been used to induce bTBI in cellular cultures, spanning a wide range of loading rates. Hydrostatic pressures have been applied to induce injury (Murphy and Horrocks, 1993; Salvador-Silva et al., 2004), but these systems do not capture the dynamics of the ICP. Systems utilizing shock tubes (Arun et al., 2011; Hue et al., 2013; Vogel et al., 2017; Campos-Pires et al., 2018) and pneumatic actuators (Ravin et al., 2012; 2016) expose cells to dynamic pressures. However, these systems are replicate the pressure from an incident shockwave—idealized as a Friedlander wave—instead of the tissue-level ICPs. Shockwaves generated by pulsed lasers (Selfridge et al., 2015; Gomez Godinez et al., 2021) and lithotripsy (Howard and Sturtevant, 1997) devices have also been utilized to induce cellular injury. However, the pressures generated are substantially more transient, in the microsecond time range, compared to the millisecond time range during blast loading.

In an interesting device design, Silvosa et al., used a piezo driven pressure chamber to control both pressure amplitude and frequency to induce primary bTBI in cerebral organoids (Silvosa et al., 2022). Approaches such as this are powerful to study mbTBI since they allow researchers to identify the relationship between specific loading parameters and injury response. Additionally, for rLLB exposures, the loading parameters require devices that are tunable to generate complex, low-magnitude pressure waveforms that are repeated for many hours (e.g., during training exercises) or at very high repetition rates to replicate firing rates of automatic weapons.

## 4 Advancing brain organoid models

Brain organoids have emerged as transformative tools for modeling human brain development and pathology, offering unprecedented opportunities to investigate complex neurobiological processes. Despite significant progress, several challenges remain for brain organoids to serve as a viable model for studying the effects of rLLB on cellular system. In this section, we review these challenges alongside recent developments aimed at addressing them.

### 4.1 Organoid growth and maturation

Due to the lack of an integrated vascular system, nutrient and waste exchange relies solely on diffusion (Nwokoye and Abilez, 2024). When organoids exceed a diameter of 0.4–0.5 mm, diffusion becomes increasingly inefficient, leading to hypoxia and necrosis of the inner core. These conditions reduce cellular viability and functional capacity in deeper regions. Additionally, brain organoids largely represent an immature state, akin to early fetal development, restricting their utility for modeling adult brain functions, such as advanced cognition or late-stage neurodegeneration. Addressing these limitations is essential for enhancing the physiological relevance and applicability of brain organoid models for mbTBI.

To overcome these challenges, various innovative techniques are being developed to support their growth and viability. One promising approach is organoid vascularization (Nwokoye and Abilez, 2024). By integrating endothelial cells into brain organoid cultures, either as a co-culture or during differentiation (Skylar-Scott et al., 2022), researchers promote vascularization, which enhances the survival of cells within the organoid’s core and promotes more complex tissue organization, closely resembling *in vivo* conditions. Vascularization is also an important component for studying neurovascular impairment, a common pathophysiology in bTBI (Siedhoff et al., 2022). Another significant advancement is the use of perfusion systems. Devices such as bioreactors and microfluidic systems enable dynamic medium flow, providing a constant supply of nutrients and oxygen while efficiently removing waste (Cai et al., 2021; Cho et al., 2021; Khan et al., 2021; Salmon et al., 2022). These systems create a more favorable microenvironment, supporting the prolonged growth and functional maintenance of larger organoids. Lastly, 3D bioprinting has emerged as a powerful tool for constructing organoids with precise spatial arrangement of cells and scaffolds, enabling the creation of vascular networks within organoids (Zhao et al., 2021; Salmon et al., 2022; Galpayage Dona et al., 2023). Together, these techniques are transforming the scalability and applicability of brain organoid models, paving the way for more advanced and realistic *in vitro* systems for studying mbTBI.

### 4.2 Cellular complexity and immune‐response modeling with microglia

One of the critical limitations of current brain organoid models is their lack of cellular diversity, which restricts their ability to replicate key processes such as neuroinflammation and immune responses to injury or disease. While some brain organoids include oligodendrocytes and myelination (Pamies et al., 2017; Chesnut et al., 2021a; Chesnut et al., 2021b)—a particularly important feature to replicate—many still lack microglia. The absence of microglia represents a significant gap in these models since they are the brain’s resident immune cell and are essential in maintaining neural homeostasis, mediating synaptic pruning, and mounting immune responses to TBI (Loane and Byrnes, 2010; Huber et al., 2016; Shi et al., 2021; Ravula et al., 2022).

To incorporate microglia into organoid systems, researchers have employed various techniques (Zhang et al., 2023). Co-culture models involve the direct addition of microglia (Abreu et al., 2018; Song et al., 2019) or iPSCs into developing organoids (Wörsdörfer et al., 2019; Fagerlund et al., 2021; Sabate-Soler et al., 2022), facilitating their interaction with other brain cell types. Alternatively, endogenous development strategies use genetic engineering or cytokine treatments to encourage microglial differentiation within the organoid itself (Ormel et al., 2018), creating a more integrated and physiologically relevant model. Emerging dynamic immune-organoid systems, enabled by microfluidic systems, further enhance this integration by allowing the interaction of circulating immune cells with organoids, simulating systemic immune response (Ramadan et al., 2023).

## 5 Non-invasive technologies

Technologies to non-invasively evaluate brain organoid responses while reproducibly increasing throughput are essential for enabling longitudinal study designs encompassing a broad parameter space. In this section, we review a range of emerging technologies that can be integrated into next-generation mbTBI platforms.

### 5.1 Microfluidic systems

High-throughput systems that integrate brain organoids with microfluidics, known as organoid-on-a-chip systems, are revolutionizing their application in research and drug discovery (Anderson et al., 2021; Zhao et al., 2024). These systems provide several advantages that enhance the scalability and control of organoid-based experiments. These systems support parallelized experiments, allowing for the simultaneous testing of multiple loading parameters, assays, intervention strategies. As discussed previously, these systems also offer precise control over critical *in vivo* factors, such as fluid flow, temperature, pH, mechanical forces, nutrient gradients, and microglia circulation, thereby creating a physiological environment that more resemble the human brain. Achieving these environments typically involves precisely controlling incubation systems and tuning media exchange using low-flow pumps to minimize shear stress. However, a unique challenge in prolonged rLLB scenarios is ensuring a robust interface between the loading device and microfluidic system. Addressing this issue is essential for the development of future mbTBI platforms. Looking ahead, several innovations promise to further enhance the utility of organoid-on-a-chip systems. The development of automated systems for the production, maintenance, and testing of organoids will streamline workflows and increase reproducibility. Additionally, enhancing integration by combining multiple organoid types (e.g., brain, liver, and heart) on a single chip will facilitate multi-organ interactions (Zhao et al., 2024), particularly relevant for pharmacokinetics and polytrauma (Hubbard et al., 2017).

### 5.2 Sensors

New multimodal sensors enable real-time monitoring of brain organoid physiology, providing researchers with continuous feedback on dynamic parameters such as mechanical properties (Ryu et al., 2021), temperature, oxygen concentration, and neural activity without disrupting the organoid (Park et al., 2021). By utilizing MEAs alongside calcium imaging, studies have demonstrated that brain organoids form neural networks that generate oscillatory activity based on phase amplitude coupling (Trujillo et al., 2019), mutual information (Alam El Din et al., 2024a), network correlation or synchrony (Samarasinghe et al., 2021; Sharf et al., 2022), which has been shown to be disrupted by bTBI (Silvosa et al., 2022). Advancements in high-density (Schröter et al., 2022) and 3D MEAs (Li et al., 2019; Soscia et al., 2020; Huang et al., 2022; Martinelli et al., 2024) are expected to drastically enhance these electrophysiological measurements through unprecedented improvements in spatial resolution and access. An emerging area called organoid intelligence (Smirnova, 2023; Smirnova et al., 2023; Alam El Din et al., 2024a; Alam El Din et al., 2024b), combines these electrophysiological measurements with artificial intelligence, opening up the possibility to study cognition, learning, and memory, all of which are effected by mbTBI (Siedhoff et al., 2022).

### 5.3 Label-free imaging

Imaging provides unique insight into the 3D structure and function of brain organoids, enabling the researchers to characterize the injury progression and recovery processes as a result of mbTBI. Confocal, multiphoton, and light sheet fluorescent microscopy are the primary techniques for 3D imaging (Ettinger and Wittmann, 2014). However, these techniques typically rely on exogenous fluorophores that are diffusion-limited, cytotoxic, or require fixation, limiting their use for long-term time-lapse imaging of brain organoids (Ettinger and Wittmann, 2014; Fei et al., 2022). Genetically engineered brain organoids that express endogenous fluorophores (Artegiani et al., 2020; Romero et al., 2023), allowing for specific tagging of processes such as oligodendrogenesis and myelination (Romero et al., 2023) have begun to address the limitations with exogenous fluorophores. However, point scanning methods such as confocal and multiphoton microscopy can induce phototoxicity (Ettinger and Wittmann, 2014), which may confound the observed effects of mbTBI.

In recent years, there has been advancements in imaging techniques that overcome these limitations by taking advantage of untagged endogenous contrast agents (Fei et al., 2022; Keshara et al., 2022; Maharjan et al., 2024). Full-field optical coherence tomography (FF-OCT) is a full-field interferometry technique that resolves the temporal dynamics of intra-cellular structures (Scholler et al., 2020; Monfort et al., 2023). This technique has been used to image retinal organoids over the course of 17 days (Monfort et al., 2023) and has been shown to be correlated with cellular processes such as oxidative stress (Groux et al., 2022), differentiation, and cellular death (Monfort et al., 2023). Techniques such as fluorescence lifetime imaging microscopy (FLIM) and hyperspectral imaging (HSI) measure properties of endogenously fluorescing biomolecules, such as decay rates and spectral characteristics, respectively, that are involved in metabolic processes, as well as structural and molecular changes in organoids (Xue et al., 2021; Barroso et al., 2023).

One of the key challenges with FF-OCT, FLIM, and HSI is achieving imaging depths beyond a few hundred micrometers (Xue et al., 2021; Monfort et al., 2023). This capability is particularly important for visualizing the inner core of organoids, which in larger brain organoids can extend to depths of 1–2 mm and may respond differently to biomechanical loading compared to the surface. Recently, three-photon microscopy (3p.m.) has been used to image cerebral organoids at depths of up to 2 mm. (Yildirim et al., 2022). The endogenous contras detected by 3p.m. is based on third harmonic generation, which is sensitive to large refractive index changes, such as those occurring at the cell membrane. However, 3p.m. is limited by the working distance of high numerical aperture (>1) immersion objectives. Recent modifications to the collection pathway are beginning to address this limitation (Deng et al., 2024). The challenge of imaging at depth is expected to become more pronounced as researchers successfully grow larger brain organoids by mitigating inner core necrosis. Consequently, further advancements in these label-free imaging techniques are necessary.

### 5.4 Sequencing

bTBI initiates a cascade of key pathophysiological processes that disrupt brain homeostasis, including excitotoxicity, oxidative stress, inflammation, and apoptosis (Siedhoff et al., 2022). These processes exacerbate the initial damage caused by the primary injury, leading to widespread neuronal dysfunction and tissue loss. Omics technologies have been extensively employed to study bTBI (Tajik and Noseworthy, 2022), providing insights into global molecular changes but failing to capture the heterogeneity of the disease. The advent of single-cell omics has addressed this limitation by enabling the investigation of responses at the level of individual cells, uncovering cell-specific biomarkers and dynamic changes in cell population distributions. For example, single-cell RNA sequencing can identify distinct transcriptional states within neurons, glial cells, and infiltrating immune cells post-injury, offering a deeper understanding of the cellular signaling driving both damage and repair across different types of external injury models (Jha et al., 2024). Additionally, when combined with CRISPR, there is a path to towards identifying and modifying injury-induced degenerative processes (Lai et al., 2024).

Although single-cell RNA sequencing is a powerful tool for precisely assessing cellular signaling pathways triggered by bTBI, it is a destructive process. Extracellular vesicle (EV) based biomarkers represent a promising alternative since nearly all cell types release EVs, making them possible to characterize by processing the supernatant in brain organoid cultures. EVs containing lipids, proteins genetic material that are reflective of the cell-type specific complex biochemical environment, enabling dynamic assessment of neuroinflammation, gliosis, and neurodegeneration (Frühbeis et al., 2013). Additionally, EVs are able to cross the blood-brain barrier (Ramos-Zaldívar et al., 2022) and hold potential for inferring the brain’s state *in vivo* (Smirnova et al., 2024).

## 6 Conclusion

Brain organoids represent a transformative technology for studying the mechanisms of mbTBI by providing a physiologically accurate *in vitro* model with unprecedented control and throughput. However, to fully realize and harness the power of these next-generation platforms, the advancement of new loading devices, organoid models, and non-invasive technologies are essential. The presented framework aims to guide research to drive these innovations, establishing brain organoids as a cornerstone in trauma research.
